# A spatially invariant noise model for minimum noise fraction (MNF) denoising of hyperspectral datasets: applications to large-scale infrared spectroscopic pathology

**DOI:** 10.1039/d6an00152a

**Published:** 2026-03-20

**Authors:** Dougal Ferguson, Peter Gardner

**Affiliations:** a Photon Science Institute, University of Manchester Oxford Road Manchester M13 9PL UK peter.gardner@manchester.ac.uk; b Department of Chemical Engineering, School of Engineering, University of Manchester Oxford Road Manchester M13 9PL UK

## Abstract

Use of Minimum Noise Fraction (MNF) denoising, previously developed for remote sensing applications, is an increasingly popular denoising technique for Infrared (IR) imaging data. The original MNF method proposed by Green *et al.* along with the faster ‘Fast MNF’ and resolution independent ‘MNF2’ all use a noise correlation matrix calculated based on neighbouring pixels, creating a heavy order-dependence. This approach fails when the spatial relationship between pixels is disrupted, for example, when large images cannot be loaded into memory on a standard workstation and are thus processed in patches or tissue data extracted using masking. We propose a spatially invariant MNF denoising method (iMNF) that uses a non-uniform, physically motivated noise estimation profile that removes this order-dependence, resulting in a robust, spatially invariant MNF based denoising algorithm. This allows for the application of the MNF denoising application to datasets where the spatial assumption is likely to be weakened by use of masking, or for unordered data such as randomly drawn labelled data, patch-wise segmentations of large scale images, or single-point spectral collections. This application was tested on representative prostate tissue biopsies for their spatial and chemical heterogeneity. Results indicate a robust, spatially invariant denoiser that is comparable to the Fast MNF method for structured and loosely structured data but is superior for unstructured data. This removes a critical bottleneck in the analysis pipeline for large IR images, such as those required in spectral pathology.

## Abstract 2

A spatially invariant version of the Minimum Noise Fraction denoising algorithm (iMNF) that removes the spatial-order dependency of standard MNF, keeping biochemical fidelity across unstructured datasets or datasets analysed in a patch-wise manner*.

## Graphical abstract

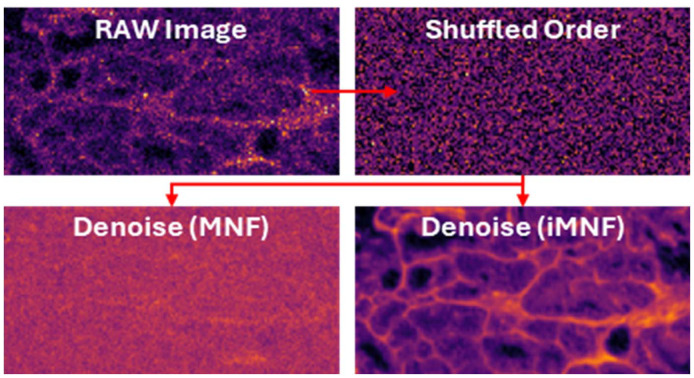

## Introduction

For IR spectroscopy, the development of focal plane array (FPA) detectors paired with increasing computational power has made the acquisition and processing of extremely large datasets possible.^1^ For many IR microscope systems, however, even with an FPA, measurement timescale requirements for large area samples make it difficult to achieve the best signal to noise ratio (SNR) for acquired data. As such, denoising techniques have become an invaluable tool to improve the quality of measured spectral data. Minimum Noise Fraction (MNF) denoising was primarily used for remote sensing applications,^2–5,16^ however given its hyperspectral format it has gained popularity in the field of IR-imaging of tissues,^6,7^ having a broad data denoising application across multiple tissue types such as prostate,^8,9^ breast,^10–12^ pancreas,^13,14^ and lymph node.^15^ MNF has been found to be one of the best performing denoisers compared to other spectral (Savitzky–Golay, Fourier transform, spectral Wavelets), spatial (Fourier transform, Mean Filter, spatial Wavelets, Deep Neural Networks), and multivariate (Principal Component Analysis) approaches for Fourier Transform Infrared (FTIR) images,^7^ showing the highest increase in classification accuracy for a supervised technique. Additionally, for the improved Quantum Cascade Laser (QCL) microscope systems, which have much improved SNR compared to FTIR, MNF denoising has been shown to improve classification results.^13^ For both FTIR and full fingerprint QCL imaging, high levels of classification accuracy were achieved when MNF was used in the preprocessing pipeline.^8^

MNF denoising is a linear transformation method that can separate signal from noise within hyperspectral data by re-projecting it into a new component space ordered by SNR, truncating the noisy components, and then transforming the data back to its original space. Similar to Principal Component Analysis (PCA), MNF uses eigenvalue decomposition to generate orthogonal components ordered by their signal content. The key difference between the two however is that PCA orders components by maximizing total variance, whereas MNF utilises a noise covariance matrix. This is particularly advantageous for spectroscopic data, where subtle chemical features of interest may exhibit low variance but high signal quality, risking their relegation to noise components in standard PCA. This noise covariance matrix (*Σ*_*δ*_) is used to decorrelate and rescale the noise (known as noise whitening). When only white-additive noise is present, MNF is equivalent to PCA denoising. This noise covariance matrix typically must be estimated from the data. As such, this noise estimation is a crucial step in the MNF application and allows for different matrix estimation techniques.^17,18^ The steps of typical MNF denoising are as follows:

First, we take a hyperspectral dataset arranged into a two-dimensional matrix *X* of *m* spectra (unrolled from the original *y* × *x* pixel dimensions) across *v* wavenumbers, where each of the *m* rows represent a single pixel's spectrum and the *v* columns represent the spectral wavenumbers.

Once arrayed, the Noise matrix *N* is estimated. In the standard implementation, this is done by calculating the difference between the spectra of adjacent pixels, with the assumption that the primary difference will be noise while the signal remains highly correlated.^2,17^ This is where MNF gets its strong order dependence.

1 *N*_*i*_ = *X*_*i*_ − *X*_*i*+1_ for *i* = 1, …, *m* − 1

It is acknowledged that unrolling the spectral data will introduce artifacts where the final pixel of a row is paired with the first pixel of the next, despite them not being spatially adjacent. However, for large hyperspectral images, these non-adjacent pairs represent a statistically negligible fraction of the total dataset. For example in a 512 × 512 pixel image, these edge transitions make up less than 0.2% of the total pixel differences calculated (511 edge transitions out of 262 143 delta calculations), meaning they have a minimal impact on the global noise covariance estimation. Similarly, while the noise covariance estimation can theoretically result in singular matrices (if spectral bands are perfectly correlated), standard implementations, including the Fast MNF used here, typically employ Singular Value Decomposition (SVD) and dimensionality reduction steps to ensure numerical stability.^19^ However, given the typical high number of spectra in hyperspectral datasets, the covariance matrices generally maintain full rank.

The covariance matrix of the estimated noise (*Σ*_*δ*_) is then calculated and diagonalized using eigenvalue decomposition.

2 *Σ*_*δ*_ = *N*^T^*N* = *VΛ*_*δ*_*V*^T^

where *V* is the matrix of the covariance matrix *Σ*_*δ*_'s eigenvectors, *Λ*_*δ*_ is the diagonal matrix of its corresponding eigenvalues. Noise whitening is then applied, where the original hyperspectral data *X* is projected into a coordinate system using the noise covariance matrix's eigenvectors and eigenvalues. The noise is transformed to have unit variance and is decorrelated across all wavenumber bands. The noise-whitened data matrix *W* is expressed as:3
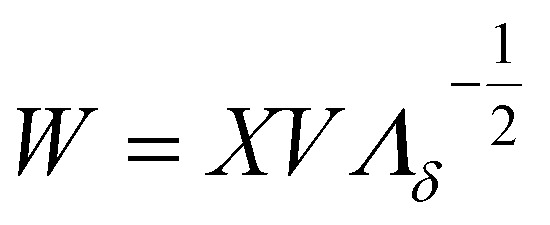


A second eigenvalue decomposition is then applied to the noise-whitened data to find a set of orthogonal components that maximise the variance. Since the noise has been normalized, ordering these components by variance is equivalent to ordering them by descending SNR.

4 *W*^T^*W* = *GΛ*_*ω*_*G*^T^

The matrix *G* represents the eigenvectors for the whitened data, and *Λ*_*ω*_ is the diagonal matrix of eigenvalues, representing the SNR of each component. The user can then select the first *K* components (referred to as bands) from *Λ*_*ω*_ that contain significant signal (the former components) and discard the rest (the latter components). This then allows for the creation of the MNF forward transformation matrix 
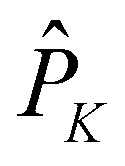
 by combining the noise whitening rotation and the principal component rotation5
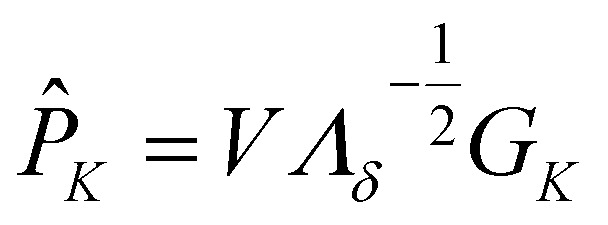


And the MNF reconstruction matrix 
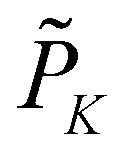
.6
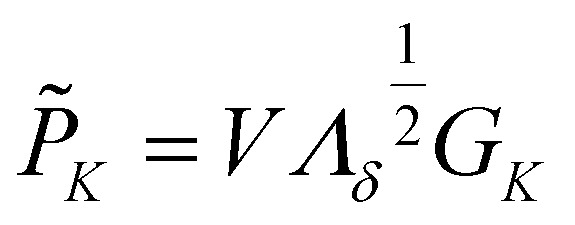


To denoise the data, the original data *X* is first projected into the truncated MNF component space, resulting in a matrix *M*_*K*_ where the components (columns) are sorted by SNR.7
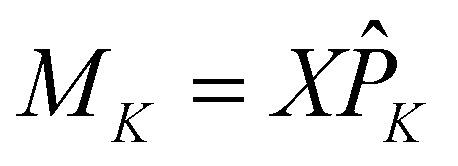


The final denoised data matrix *D* is then calculated by projecting *M*_*K*_ using the transpose of the reconstruction matrix.8
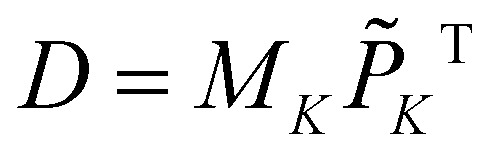


Requiring the data to be ordered for effective noise array estimation restricts this method to image arrayed data only, which while suitable for whole hyperspectral images, may not be apt for new hyperspectral analysis techniques.

**Fig. 1:**
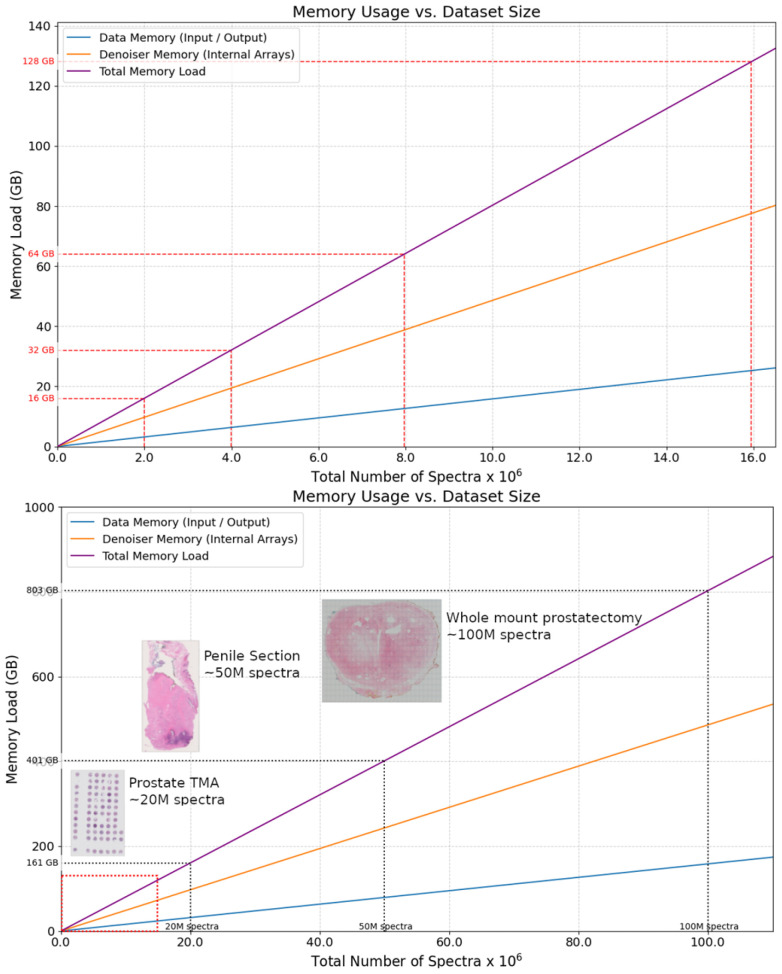
Computational demands of the standard MNF workflow for progressively larger hyperspectral datasets. The increase in memory required for both raw input data (blue solid line), the MNF denoising arrays (orange line), and full memory load (purple solid line) for full fingerprint spectra captured at 4 cm^−1^ spatial resolution with 2 cm^−1^ step sizes (totalling a [1, 425] size array per spectrum) (top) is plotted against the capacity of standard computing equipment memory (16, 32, 64, and 128 GB RAM, red dotted lines). Total spectra for three different sample types (a prostate tissue micro array, penile section, and whole mount prostate section) are provided (bottom) to highlight the key limitation of data load for large samples, the scale at which we surpass the 128 GB memory limitation is highlighted (dotted red square).

While the time to load and denoise large hyperspectral images remains low, most standard computing equipment cannot handle the loading and denoising of datasets with more than 10 million spectra in the fingerprint region (950–1800 cm^−1^), as illustrated in Fig. 1. This amount of memory load makes it infeasible to perform standard MNF denoising on large tissue structures, for example a 28 × 18 mm^2^ scan region of a typical tissue micro array area can contain ∼20 million spectra when scanned using a QCL spectrometer. This number is further increased for larger tissue sections such as penile or whole mount prostatectomy samples, which can more than double the region of tissue required for scanning.^8^

Alternative noise estimation methods exist, such as the ‘two-scan’ approach that directly measures noise by differencing two consecutive images. However this is impractical for large-area mapping as the method doubles the total data volume, exacerbating the memory and storage challenges already highlighted.^20^ A common strategy to circumvent such memory constraints would be to perform patch-wise denoising, where smaller patches of data are denoised independently and then stitched together. The memory requirements for the implementation of patch-wise denoising (assuming a 128 by 128 patch) for the key tissue samples outlined in Fig. 1 are calculated in Table 1.

**Table 1:** Memory usage comparison for applying the standard MNF denoising algorithm on the key tissue samples outlined in Fig. 1 against applying a patch-wise MNF algorithm treating 16 384 spectra at a time (128 × 128 pixels)

Total spectra	Full image method (GB)	Patch-wise method (GB)	Memory saving (%)
20m	160.6	33.7	79.0%
50m	401.4	81.8	79.6%
100m	802.8	161.9	79.8%

**Fig. 2:**
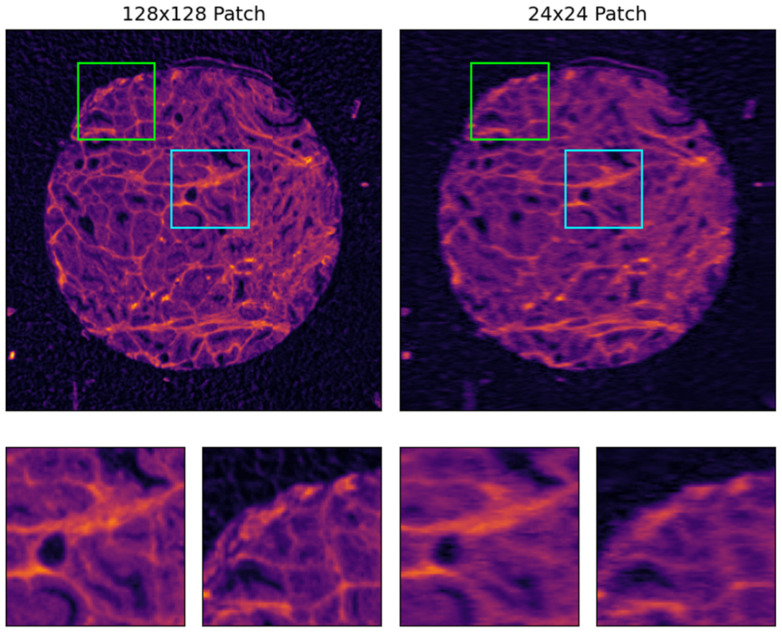
A comparison of patch-wise MNF denoising applied to a representative prostate tissue core at the Amide I band (∼1656 cm^−1^), with patch sizes of 128 × 128 (left) compared against 24 × 24 (right). The patch size is shown to heavily influence the resultant denoising capabilities of the MNF algorithm.

While the memory savings in Table 1 are substantial, the total memory for these datasets still exceed standard computing limits. It should be noted that these values represent holding the entire output image in memory; a dynamic write-to-disk workflow that processes and saves each patch directly to storage would virtually eliminate this final memory constraint. However, such a patch-wise approach introduces a more fundamental conceptual flaw for standard MNF denoising. Given that the MNF algorithm estimates noise based on the data provided, each patch will therefore generate its own unique, and possibly inconsistent, noise model. The reliance on localized context windows can lead to significant image degradation requiring hyperparameter optimisation, which can be unsuitable for automated pipelines. Fig. 2 illustrates the application of a patch-wise approach, showing the impact of differing patch sizes based on those used in prior literature.^8,19–21^

**Fig. 3:**
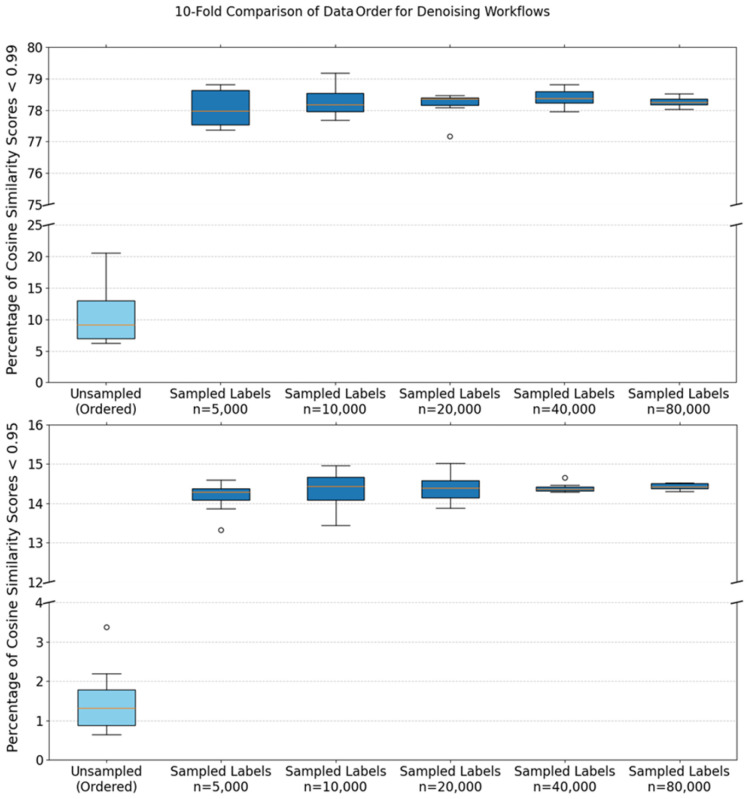
Quantitative comparison of the similarity difference between ordered and sampled datasets across 10 different folds of calculations. The plots show the distribution of the percentage of adjacent spectra with a cosine similarity below high-correlation thresholds of 0.99 (top) and 0.95 (bottom) across each set of data. For training sets compiled from randomly sampled labels, the percentage of dissimilar pairs is dramatically higher, irrespective of the total number of samples.

Given the inherent issues with a patch-wise workflow, one might consider an entirely different approach to the memory problem: masking away irrelevant data (such as paraffin wax), and sample from known labelled data to build an “ordered” array of spectra that consist of tissue types expected in our samples from which to estimate this noise covariance matrix. However, even when sampling labelled data of known tissue types, the order dependency assumption is flawed. To test this, the cosine similarity of adjacent pixels is compared between the whole dataset of 10 different ordered samples, and 10 different sets of sampled labelled data at varying levels of sampled spectra (from 5000 to 80 000 spectra). As illustrated in Fig. 3, the cosine similarity is very different between ordered data and sampled data, irrespective of the sample size. The percentage of adjacent pixels with a cosine similarity below a 0.99 threshold (a very high degree of correlation) increases from ∼15% to ∼75% when sampling data, with over a 7-fold average increase in cosine similarity values below 0.95, highlighting a key flaw in the current MNF denoising algorithm.

Taking these issues into consideration, we decided to design a new spatially invariant noise estimation method, herein referred to as iMNF, based on the analysis of spectral silent regions to calculate the noise covariance matrix. This physically motivated noise estimation profile removes this order-dependence of neighbouring pixel differencing.

Instead of eqn (1), we first calculate a single base noise variance (*σ*^2^_base_) which is calculated as the average column variances of the first derivative (using a Savitzky–Golay first derivative filter) of a user defined spectral region (*X*_d*z*_). The derivative is calculated using a Savitzky–Golay (SG) filter with a window length of 5 points and a 2nd-order polynomial, chosen to provide a stable noise estimate without being overly sensitive to minor spectral variations. A parameter sweep was conducted to confirm modelling stability, which showed negligible variation in the denoised output across a range of window lengths and polynomial orders (SI, Fig. S1).

9 *σ*^2^_base_ = mean(Var(*f*_SG′_(*X*_d*z*_)))

It should be noted that the application of the SG first derivative acts as a high-pass filter. In the silent region, the underlying chemical signal is expected to be constant or varying slowly, while noise is expected to manifest as rapid, high-frequency fluctuations. The derivative operation supresses the low-frequency baseline signal, ensuring that any calculated variance is attributable to noise. While the magnitude of this derivative variance differs from the raw noise variance by a scalar factor determined by the filter coefficients, this scalar scaling does not affect the performance of the MNF transform. The MNF rotation is determined by the eigenvectors of the noise covariance matrix; a global scalar multiplier scales the resulting eigenvalues (SNR) but preserves the eigenvectors and the relative ordering of components.

We choose a region that is within the generally biologically silent region of the spectrum, for QCL data this is 1800–1750 cm^−1^ (with 1950–1750 cm^−1^ possible) and 2200–1750 cm^−1^ for FTIR data. This region provides a representative estimate of the global noise present throughout the entire dataset. Note that although some biological systems might contain frequencies from the C=O stretching functional group in the 1800–1750 cm^−1^ region,^22–24^ they are generally not observed in tissue and are expected to be consistent enough to not be calculated as noise following the derivatization step. Furthermore, the derivatization step helps mitigate the influence of broad underlying spectral features, ensuring that only high-frequency fluctuations contribute to the noise estimation. While the choice of the silent region can be performed on user inspection, this is not functional for automated analytical pipelines. To address this and ensure robustness against atypical chemistry, an integrated automated window-selection heuristic can be performed within the iMNF algorithm. A sliding window is passed over the silent region to identify the region of lowest baseline first-derivative variance. Furthermore, an additional Quality Control (QC) step is applied to check for atypical chemistry. The presence of narrow-band contaminants can be assessed by checking for localised variance spikes, removing the need for user visual inspection for silent region range selection.

We then construct the noise covariance matrix *Σ*_*δ*_ using a physically accurate non-uniform scaling protocol. This scales the base noise variance based on the principle that noise variance in absorbance units is inversely proportional to the square of transmittance. While instrument response and detector noise regimes are not perfectly constant across the spectrum, the primary driver of signal-dependent noise in both QCL and FTIR absorbance data is the logarithmic transformation of transmittance. As transmittance approaches zero (regions of high absorbance), this mathematical conversion non-linearly amplifies the inherent instrumental baseline noise. This scaling approach provides a robust, first-order approximation of this dominant effect, generating a more realistic noise profile across the full spectral range. To do this we start by computing a mean absorbance spectrum (
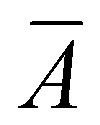
) and converting it to a mean transmittance spectrum (
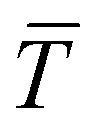
).10
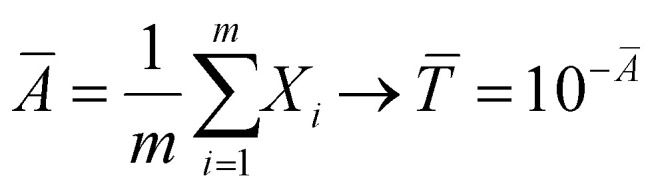


A reference transmittance value (
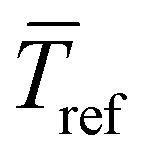
) is calculated by averaging the mean transmittance spectrum within the user specified region (the silent region)11
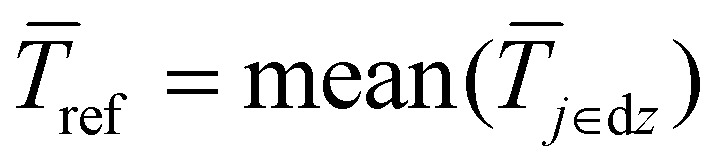
where *j* represents the indices of the wavenumbers in the specified region (d*z*). We then compute a scaling factor (*S*) based on the relationship between the reference transmittance and the transmittance at every wavenumber. This factor is used to scale the base noise, creating a non-uniform noise profile vector (*σ*^2^_profile_).12
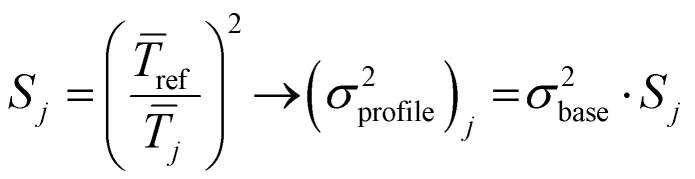


The final noise covariance matrix (*Σ*_*δ*_) is then constructed as a diagonal matrix using the non-uniform noise profile vector.

13 *Σ*_*δ*_ = diag(*σ*^2^_profile_)

By constructing *Σ*_*δ*_ strictly as a diagonal matrix, we explicitly position the iMNF as a robust diagonal approximation. While standard IR detectors often exhibit highly correlated noise across wavenumbers, this formulation intentionally ignores those off-diagonal inter-wavenumber correlations. This trade-off is the specific mechanism that grants iMNF its spatial invariance. This resulting diagonal matrix, *Σ*_*δ*_, represents a global noise profile for the entire dataset. Crucially, as its calculation depends only on the mean spectral properties and not on the differences between adjacent pixels, it is completely spatially invariant, directly addressing the order-dependency of the standard model derived from eqn (1). From here all previous steps for MNF denoising are followed. Furthermore, this singular global matrix can be calculated once and uniformly applied to any subsequent data subsets, avoiding inconsistent noise modelling issues that could impact patch-wise denoising workflows.

This modification to the noise covariance matrix estimation removes any order dependency of the data for the application of the MNF denoising process, without requiring any change to the data acquisition process of IR data.

To validate the performance of the iMNF approach, we compared the effect of applying fastMNF (referred to as “standard” MNF) to the iMNF for several scenarios on a prostate cancer tissue biopsy core. The primary analysis was conducted on data from a modern QCL instrument (Bruker LUMOS II ILIM), with all experiments replicated on an FTIR dataset (Agilent Cary 600 series) for verification. The biopsy core was chosen due to its heterogeneity with numerous tissue constituents: stroma, epithelium, blood, corpora amylacea. Comparisons will be made to highlight iMNF's ability to handle spatial invariance of shuffled data, the denoising performance of extracted spectra in ordered and unordered fashion, and the denoising robustness when employing tissue extraction before denoising.

## Materials and methods

### Tissue collection

Human prostate tissue samples were obtained from the Manchester Cancer Research Cancer Biobank (10_NOCL_02) from tissue collected following ethical approval by the South Manchester Research Ethics Committee (Ref: 22/NW/0237). Formalin-fixed paraffin-embedded prostate tissue samples from patients undergoing transurethral resection of the prostate (TURP) or transrectal ultrasound guided needle core biopsies (TRUSBx) between 1994 and 2004 were used to construct tissue micro arrays (TMAs). Tissue sections were cut at 5 μm and loaded onto calcium fluoride (CaF_2_) slides for spectral imaging.

### QCL imaging

QCL data was acquired using a Bruker LUMOS II ILIM, with a 520 × 480 focal plane array (FPA) room temperature microbolometer and a refractive 4× IR objective lens (0.6 NA). This system has a 2.2 by 2 mm^2^ field of view (FOV) and a nominal pixel size of 4.3 by 4.3 μm^2^. The instrumental optics were purged with dry air and despite the sample compartment having an open design, no significant water vapour bands were detected in the recorded spectra. A single background scan was collected from an area of clean paraffin-free calcium fluoride (CaF_2_) for each slide, respectively. Full spectra were recorded in sweep scan mode within a spectral range of 1800 to 952 cm^−1^ using a spectral sampling interval of 2 cm^−1^ without co-added scans (*i.e.*, one scan per sampling area).

### FTIR imaging

FTIR scans of the tissues were acquired using an Agilent Cary 670-IR spectrometer coupled to an Agilent Cary 620-IR imaging microscope, equipped with a liquid nitrogen cooled mercury cadmium telluride (MCT) focal plane array (FPA) detector with 128 × 128 detector elements. A 15× Cassegrain microscope objective was used producing a field-of-view measuring 704 × 704 μm^2^, with each pixel measuring 5.5 × 5.5 μm^2^. A sealable enclosure covered sample stage and optics, providing a continuous supply of dry air. Data were collected with a humidity level <1% to remove any water vapour from the compartment that could have been recorded as part of the spectrum. Background scans, collected from a section of clean paraffin-free calcium fluoride (CaF_2_), consisted of 256 co-additions at a spectral resolution of 5 cm^−1^. For tissue scans images were obtained as a mosaic of multiple tiles, each with 8 co-added scans. Blackmann–Harris interferogram apodisation was used with two levels of zero filling, with a spectral range of 900 to 3800 cm^−1^.

### Data pre-processing

All calculations were performed in Python (version 3.9). The code for the standard MNF algorithm applied in the study was based on the original MATLAB code presented by Gupta *et al.*^19^ The iMNF noise estimation code was written inhouse and can be accessed *via* Zenodo (https://doi.org/10.5281/zenodo.17092326).^25^ Unless otherwise stated, all denoising was performed with 30 bands for reconstruction. This threshold was selected in accordance with established spectral pathology literature.^8,19^

For supervised classification tasks, QCL and FTIR data underwent the following common preprocessing steps:

- Linear baseline subtraction.

- Denoising step (30 bands for PCA, MNF, iMNF denoising, window length 13 polynomial order 2 for Savitzky–Golay smoothing, and sym4 wavelet denoising with level 3).

- Truncation to fingerprint region (1800–1000 cm^−1^).

- Removal of wax regions (1490–1360 cm^−1^).

- Vector normalisation.

- 2^nd^ derivative conversion (19 window size, 4^th^ order polynomial).

To confirm iMNF parameter stability (specifically the window length and polynomial order of the base noise variance matrix *σ*^2^_base_), a parameter sweep was performed to show negligible variation in the denoised output across window lengths 5 to 11 and polynomial orders of 2 to 4 (SI Fig S1).

Silent regions for iMNF denoising for QCL (1750–1800 cm^−1^) and FTIR (1750–2200 cm^−1^) were chosen following the analysis of baseline derivative variance, choosing the region with the lowest derivative variation.

## Results

**Fig. 4:**
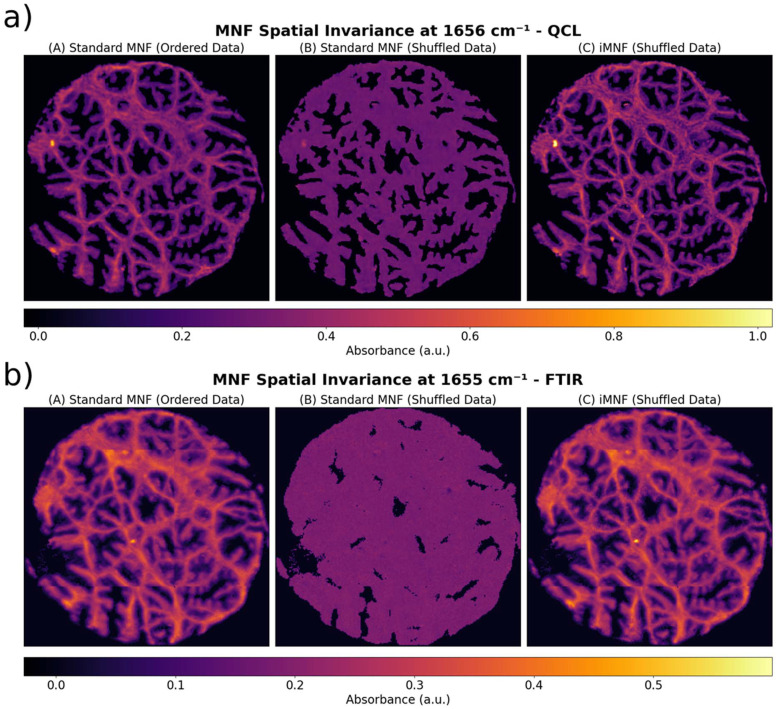
Demonstration of spatial invariance. A comparison of denoising performance at ∼1656 cm^−1^ on a prostate tissue core imaged using a QCL microscope (a) and an FTIR microscope (b). (A) The denoised image produced by standard MNF on the original, spatially ordered data. (B) The result of applying standard MNF to the same data after randomly shuffling the pixel order, demonstrating a complete failure of the algorithm. (C) The result of the proposed iMNF method on the shuffled data.

A key benefit of the iMNF approach is its spatial invariance, allowing the denoising step to not be affected by any change to data order. The influence of data shuffling is compared visually by plotting the spatial distribution of the Amide I band (∼1656 cm^−1^) in Fig. 4, where the standard application of MNF denoising on ordered and shuffled data across QCL and FTIR modalities is compared against the application of iMNF denoising on shuffled data. The inclusion of FTIR data, which possess different inherent noise characteristics and baseline signal intensities compared to QCL, further validates the robustness of iMNF across IR modalities. The data denoised using the iMNF approach is still spatially structured after reordering, whereas the standard MNF method fails to reproduce the same results. The fact that the standard MNF application on the shuffled data results in a uniform featureless image directly contrasts with the successful iMNF method, highlighting this key feature of spatial invariance.

**Fig. 5:**
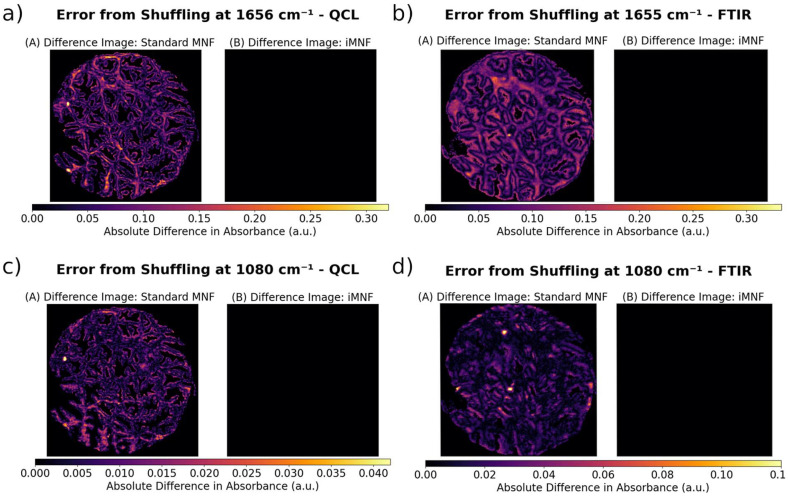
Pixel-wise absolute difference maps for QCL data illustrating the error introduced by shuffling the data order before denoising. The difference is calculated between the denoised result from ordered data and the denoised result from shuffled data across data captured at ∼1656 cm^−1^ and 1080 cm^−1^ wavenumber on a QCL microscope (a and c respectively) and an FTIR microscope (b and d respectively). (A) The error calculated between ordered and shuffled data for the standard MNF approach. (B) The error calculated between ordered and shuffled data for the proposed iMNF approach.

A more quantitative comparison is to calculate the pixel-wise error introduced by shuffling the data for each of the methods, as presented in Fig. 5. The corresponding difference maps for the FTIR dataset, shows the same outcome. The standard MNF approach shows significant error across the entire tissue structure, indicating it is not robust to order changes. The difference image for the standard MNF application highlights the reliance on pixel order; shuffling the data introduces errors because the “adjacent” pixels are no longer physically close, leading to inaccurate noise estimation and a distorted noise image. In contrast, the iMNF difference image is zero across the image, demonstrating that the results of iMNF denoising is spatially invariant.

**Fig. 6:**
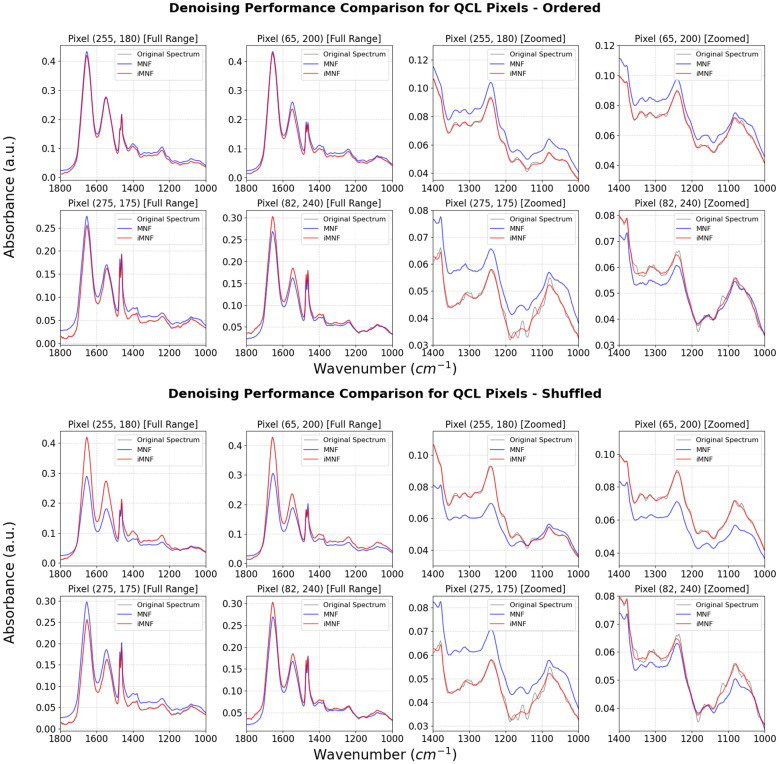
A comparison of denoising performance on representative spectra from tissue pixels for the QCL imaged tissue. The top row shows results for spatially ordered data, while the bottom row shows results for randomly shuffled data. Each plot compares the original spectrum (grey) with the results from standard MNF (blue) and iMNF (red).

**Fig. 7:**
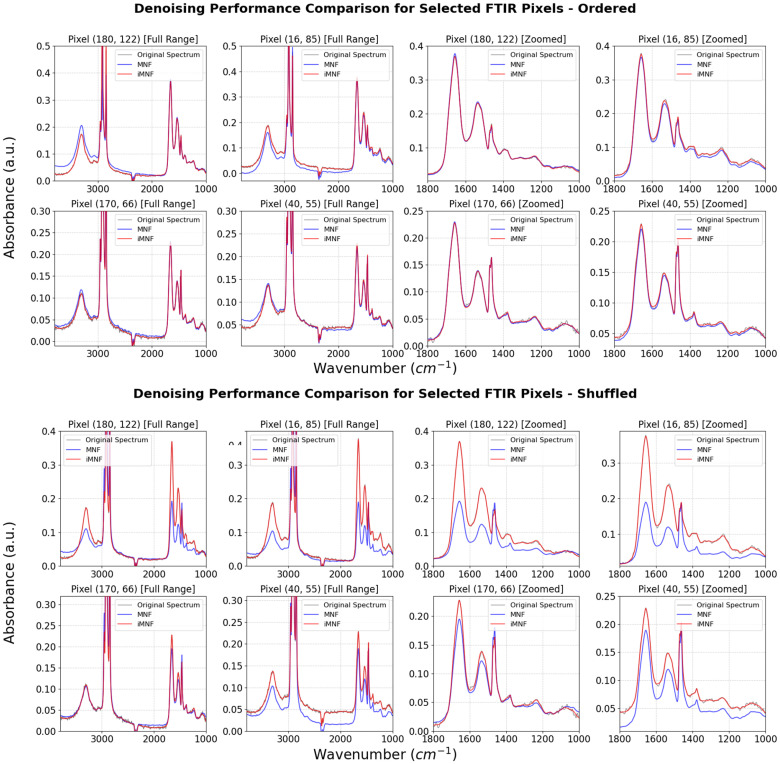
A comparison of denoising performance on representative spectra from epithelial tissue pixels for the FTIR imaged tissue. The top row shows results for spatially ordered data, while the bottom row shows results for randomly shuffled data. Each plot compares the noisy spectrum (grey) with the results from standard MNF (blue) and iMNF (red).

To fully confirm the model's applicability, the spectral profiles of denoising pixels must be compared. In prostate tissue cores, most of the tissue will either be stromal or epithelial. As such, multiple spectra from each of these groups were plotted for comparison in Fig. 6 for QCL and Fig. 7 for FTIR data respectively. An important finding is that for ordered data, MNF and iMNF produce comparable denoising results. This comparison serves as a benchmark against the standard, correct application of the MNF algorithm, demonstrating that iMNF achieves equivalent performance to the established method when spatial assumptions hold true. To quantify the similarity, pixel-wise cosine similarity between the outputs of both models was calculated, confirming a very high degree of spectral similarity with a mean score of 0.9996 (SI, Fig. S2). However for unordered data, standard MNF produces distorted results, whereas the iMNF continues to provide effective denoising.

**Fig. 8:**
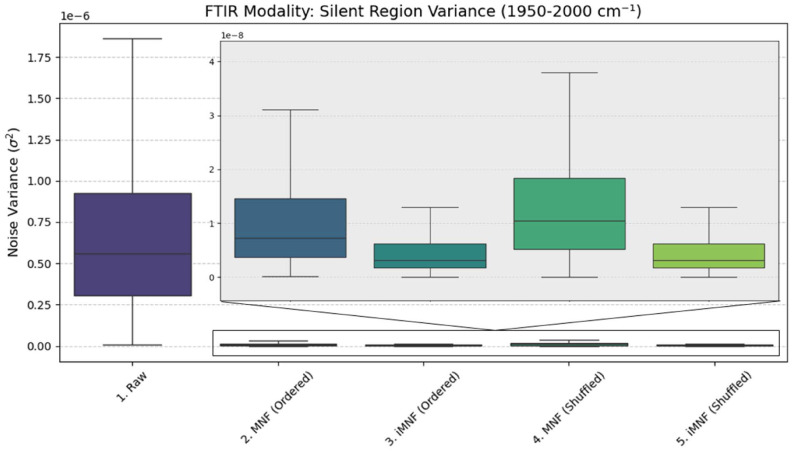
Quantitative comparison of absolute noise variance reduction within the silent region (1950–2000 cm^−1^) across FTIR imaged prostate tissue cores. Box plots display the baseline noise variance of raw (unprocessed) data compared to the standard MNF and iMNF applied to both spatially ordered (full image) and spatially shuffled (unstructured) data arrays.

**Fig. 9:**
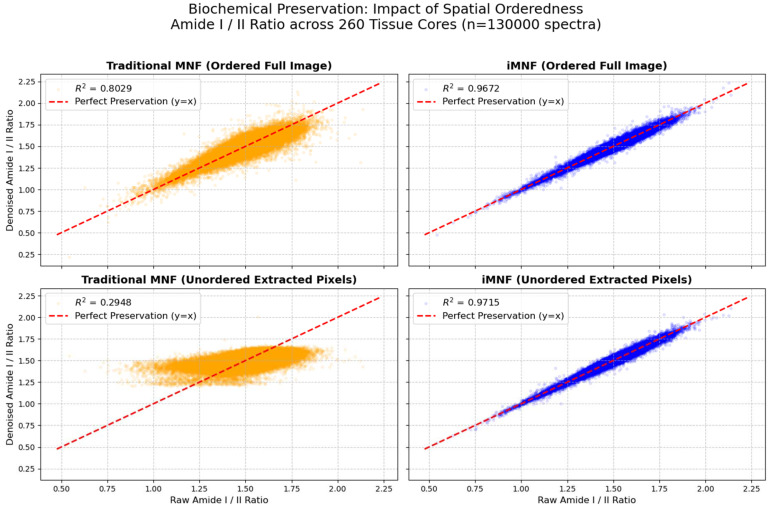
Assessment of biochemical preservation on Amide I/Amide II ratios across a subsample of spectra (*n* = 130 000) across 260 FTIR imaged tissue cores. Standard MNF and iMNF were applied to spatially ordered full images (top) and unstructured shuffled pixels (bottom). Biochemical preservation was assessed by comparing the denoised ratios to the raw data ratios using *R*^2^ correlation.

To evaluate biochemical preservation and SNR improvements, analysis was extended to a cohort of 260 FTIR-imaged prostate tissue cores. Firstly, the absolute variance reduction in the biochemically silent region (1950–2000 cm^−1^) was assessed as a proxy for noise removal capacity, as shown in Fig. 8. Whether applied to ordered or shuffled, unstructured data, iMNF maintains strict spatial invariance, replicating its noise reduction irrespective of data order. Additionally, to quantify the preservation of chemical features, the impact of denoising on the Amide I/Amide II ratio across 130 000 extracted spectra from the same cohort was performed. When applied to complete, spatially ordered data, both standard MNF and iMNF demonstrate strong preservation of the biochemical ratios (*R*^2^ = 0.8 and *R*^2^ = 0.97 respectively), shown in Fig. 9. However, when the algorithm is applied to unstructured patch arrays of extracted pixels (of size 500 pixels per patch), the algorithms diverge. Standard MNF fails to construct an accurate noise model without spatial adjacency, resulting in strong distortion of the Amide I/Amide II ratio (*R*^2^ = 0.29), while iMNF retains its strong preservation (*R*^2^ = 0.97), regardless of the data structure.

The impact of the denoising methods on downstream ML tasks was also analysed. A 10-fold patient-isolated cross-validation using a Random Forests classifier was performed to assess the capacity to improve simple tissue classification of prostate tissues (epithelium, stroma, immune cell infiltration, corpora amylacea, and red blood cells) on a small subset of balanced data (10 000 spectra per group) across both QCL and FTIR modalities, with results presented in Table 2. These techniques were compared against standard spectral smoothing approaches (Savitzky–Golay filter and Wavelet smoothing). To accurately simulate the breakdown of spatial order required by standard dataset partitioning, the extracted spectral arrays were subjected to the default random shuffling approach employed by the scikit-learn library prior to denoising. While standard MNF applications have been shown to achieve high classification accuracy on ordered hyperspectral images,^8^ the explicit breaking of the spectral orderedness assumption results in a breakdown of the algorithm, severely degrading the MNF specific modelling results.

**Table 2:** Downstream machine learning performance for a 10-fold patient isolated cross-validation using a Random Forests classifier for key prostate tissue constituents (epithelium, stroma, immune cell infiltration, corpora amylacea, and red blood cells) from highly unstructured, randomly shuffled data arrays. Performance is compared across non-denoised (raw), PCA, standard smoothing functions (Savitzky–Golay and Wavelets), standard MNF, and iMNF across QCL and FTIR data modalities

Class	Track	QCL 10-fold cross validation average (std. dev. ≥0.01)				FTIR 10-fold cross validation average (std. dev. ≥0.01)			
		Sensitivity	Specificity	Precision	F1-score	Sensitivity	Specificity	Precision	F1-score
Epithelium	RAW	0.896 (0.03)	0.864 (0.05)	0.646 (0.09)	0.746 (0.04)	0.850 (0.04)	0.842 (0.05)	0.687 (0.08)	0.756 (0.04)
	PCA	**0.912 (0.03)**	0.907 (0.04)	0.730 (0.09)	0.808 (0.05)	0.901 (0.03)	0.817 (0.05)	0.670 (0.07)	0.769 (0.05)
	SG	0.896 (0.03)	0.874 (0.04)	0.663 (0.08)	0.758 (0.04)	0.867 (0.04)	**0.845 (0.05)**	**0.697 (0.08)**	0.768 (0.03)
	Wavelet	0.897 (0.03)	0.870 (0.04)	0.657 (0.09)	0.754 (0.04)	0.861 (0.04)	0.844 (0.05)	0.694 (0.08)	0.764 (0.03)
	MNF	0.277 (0.25)	0.724 (0.25)	0.215 (0.03)	0.199 (0.09)	0.537 (0.18)	0.489 (0.18)	0.298 (0.03)	0.370 (0.04)
	**IMNF**	0.909 (0.03)	**0.913 (0.03)**	**0.741 (0.08)**	**0.814 (0.04)**	**0.953 (0.01)**	0.827 (0.05)	0.692 (0.09)	**0.799 (0.06)**
Stroma	RAW	0.904 (0.02)	0.982	0.931 (0.01)	0.917 (0.01)	0.895 (0.02)	0.897 (0.03)	0.776 (0.05)	0.823 (0.03)
	PCA	0.949 (0.02)	**0.987**	**0.953**	**0.951**	0.896 (0.02)	0.940 (0.02)	0.857 (0.04)	0.875 (0.02)
	SG	0.912 (0.02)	0.982	0.933	0.922 (0.01)	0.909 (0.02)	0.919 (0.02)	0.817 (0.05)	0.859 (0.03)
	Wavelet	0.912 (0.02)	0.982	0.932	0.922 (0.01)	0.907 (0.02)	0.912 (0.02)	0.805 (0.05)	0.852 (0.03)
	MNF	0.180 (0.16)	0.833 (0.15)	0.225 (0.01)	0.156 (0.12)	0.370 (0.17)	0.656 (0.16)	0.303 (0.03)	0.315 (0.08)
	**IMNF**	**0.951 (0.01)**	**0.987**	0.952	**0.951**	**0.941 (0.01)**	**0.969**	**0.923 (0.02)**	**0.932 (0.01)**
Immune infiltration	RAW	0.599 (0.11)	**0.986**	0.892 (0.07)	0.709 (0.07)	0.103 (0.18)	0.982 (0.02)	0.311 (0.11)	0.103 (0.11)
	PCA	0.680 (0.13)	**0.986**	0.907 (0.04)	0.769 (0.09)	0.011 (0.02)	**0.998**	0.218 (0.22)	0.021 (0.03)
	SG	0.623 (0.11)	**0.986**	0.896 (0.06)	0.728 (0.07)	**0.122 (0.19)**	0.981 (0.02)	0.401 (0.18)	**0.129 (0.10)**
	Wavelet	0.610 (0.12)	**0.986**	0.897 (0.06)	0.719 (0.07)	0.118 (0.19)	0.982 (0.02)	0.382 (0.18)	0.120 (0.10)
	MNF	0.006 (0.02)	0.994 (0.02)	0.106 (0.19)	0.009 (0.03)	0.015 (0.02)	0.986 (0.02)	0.190 (0.29)	0.021 (0.03)
	**IMNF**	**0.700 (0.12)**	0.985	**0.906 (0.04)**	**0.783 (0.08)**	0.064 (0.13)	**0.998**	0.611 (0.20)	0.095 (0.16)
Corpora amylacea	RAW	0.954 (0.03)	0.997	0.990	0.971 (0.01)	0.953 (0.03)	**0.998**	0.990	0.971 (0.01)
	PCA	0.972 (0.01)	**0.998**	**0.992**	**0.982**	0.980 (0.01)	0.997	0.986	0.983
	SG	0.959 (0.03)	**0.998**	0.991	0.975 (0.01)	0.967 (0.02)	**0.998**	0.990	0.979
	Wavelet	0.959 (0.03)	0.997	0.990	0.974 (0.01)	0.964 (0.02)	**0.998**	0.990	0.977 (0.01)
	MNF	0.294 (0.31)	0.719 (0.30)	0.213 (0.03)	0.184 (0.13)	0.134 (0.10)	0.898 (0.08)	0.267 (0.11)	0.150 (0.07)
	**IMNF**	**0.974 (0.01)**	0.997	0.990	**0.982**	**0.983 (0.01)**	**0.998**	**0.991**	**0.987**
Red blood cells	RAW	0.925 (0.06)	**0.999**	**0.995**	0.958 (0.03)	0.850 (0.07)	0.996	0.958 (0.03)	0.900 (0.04)
	PCA	**0.969 (0.02)**	**0.999**	**0.995**	**0.981 (0.01)**	0.920 (0.03)	0.994	0.948 (0.04)	0.933 (0.02)
	SG	0.931 (0.05)	**0.999**	**0.995**	0.961 (0.03)	0.885 (0.05)	0.996	0.961 (0.02)	0.920 (0.02)
	Wavelet	0.930 (0.05)	**0.999**	**0.995**	0.960 (0.03)	0.875 (0.05)	0.996	0.963 (0.02)	0.916 (0.03)
	MNF	0.261 (0.35)	0.735 (0.34)	0.257 (0.27)	0.139 (0.15)	0.010 (0.02)	0.991 (0.02)	0.137 (0.11)	0.015 (0.02)
	**IMNF**	0.968 (0.02)	**0.999**	**0.995**	**0.981 (0.01)**	**0.935 (0.02)**	**0.997**	**0.971 (0.02)**	**0.953 (0.01)**

**Fig. 10:**
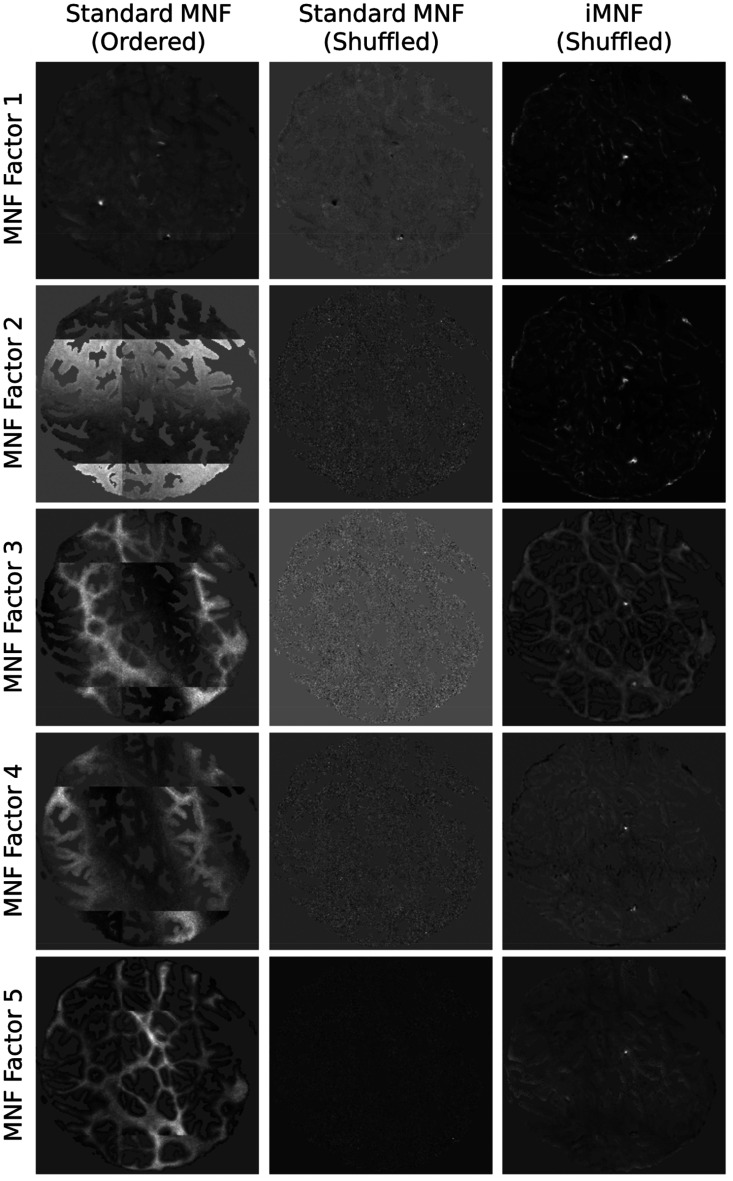
Comparison of MNF factor images processed under three conditions: standard order-dependent MNF applied to the adulterated noisy data (left column). The same standard MNF method applied to same data but spatially shuffled (middle column). The proposed order-independent iMNF method applied to the spatially shuffled noisy data (right column).

Lastly, comparing the MNF factors themselves can provide additional visualization of the denoising process for each method. MNF factors are ordered by SNR, where the first few factors are the largest eigenvalues which contain the bulk of coherent signal information and key morphological features. Therefore, we expect to see clear delineated tissue structures in early components if the model is working as intended. As shown in Fig. 10, the MNF factors for the standard method for ordered data show well-defined tissue morphology, whereas the same method applied to shuffled data appears to show very little morphology in any of the top factors. In contrast, the iMNF method applied to the same shuffled data is clearly showing underlying tissue morphology. The ability to identify and preserve key morphological signal even with shuffled data indicates a robustness for unordered datasets.

## Discussion

A key strength of the proposed iMNF method is its ability to overcome the order-dependency of standard MNF by employing a spatially invariant, global noise model. However, it is important to note the inherent assumptions and trade-offs involved in this design. Standard IR detectors exhibit highly correlated noise across wavenumbers due to detector/readout characteristics, baseline shifts, thermal drifts, optical fringing, and (specific to QCL) acquisition and sweep-related effects. To quantify this, the noise covariance matrix of the silent region is empirically estimated using adjacent-pixel differencing on the ordered image datasets presented in this study. Inter-wavenumber noise correlation was found to be very high (mean absolute off-diagonal correlations of 0.985 for FTIR [1750–2200 cm^−1^] and 0.987 for QCL [1750–1800 cm^−1^]).

Standard MNF attempts to model these complex off-diagonal correlations by deriving the covariance matrix from spatial adjacencies, creating a strict ordered dependency. This local estimation collapses when data is unordered, as demonstrated by the downstream classification benchmarks, implying the standard algorithm misinterprets non-adjacent biochemical variance as correlated noise.

Therefore, iMNF is explicitly formulated as a robust diagonal approximation. By enforcing a diagonal covariance matrix (eqn (13)), the algorithm is purposefully ignoring local inter-wavenumber noise correlations, making it spatially invariant. The expected failure mode of this diagonal approximation comes from unmodelled correlated noise. As iMNF ignores the off-diagonal terms, highly correlated noise structures, such as severe optical fringing, will not be perfectly whitened during the transformation. Consequently, in datasets where spatial order is perfectly preserved and such correlated noise is prominent, standard MNF will likely achieve better denoising results.

However, by discarding this local context, iMNF relies instead on the global properties of the dataset, specifically the biochemically “silent region” and the mean transmittance spectrum. While this makes the algorithm robust and powerful to unordered data, it is inherently agnostic to the local neighbourhood of any given pixel. This distinction highlights the core difference between the two approaches: standard MNF excels at capturing context specific noise, while iMNF provides a universally applicable, order-independent noise model.

Therefore, for datasets where spatial order can be guaranteed, either by using sufficient computational resources to process a large image in its entirety or by subsampling complete, contiguous regions, the standard MNF approach remains a powerful, and potentially superior, tool for capturing context-specific noise, as shown in prior classification works.^8^

The strict reliance on spatial order in standard MNF applications inherently limit its application if the user is not considerate of its pitfalls, especially for complex and heterogeneous datasets. For large hyperspectral images of tissues, the spectra of interest (the tissue components) are typically extracted from the hyperspectral image due to hardware memory limitations restricting the user from keeping all measured data. This reduces the ordered assumption of the spectral data, limiting the effectiveness of the MNF method. The breakdown of spatial adjacency is particularly problematic for histopathological samples, which are often highly heterogenous and morphologically complex. For instance, when analysing a tissue biopsy, the process of masking out non-tissue background can leave behind discontinuous regions of interest, such as cancerous and non-cancerous epithelium, stroma, and necrosis. In the resulting data array, “adjacent” pixels may be pixels located across a void or belonging to a different tissue type such as blood vessels, creating a difference value dominated by biochemical variance rather than noise, creating an inaccurate noise covariance estimation. Furthermore, this strict reliance on spatial order inherently limits the application of conventional MNF to imaging datasets alone. This precludes its use for other valuable forms of spectral data, such as collections of single-point scans or other unstructured datasets where a PCA-like analysis is desired but could be significantly enhanced by a more sophisticated noise model.

## Conclusions

In this work we have addressed a fundamental limitation of the Minimum Noise Fraction (MNF) denoising method: its dependence on spatial order. This approach relies on the assumption of local similarity between adjacent pixels, which is ineffective for unstructured or spatially discontinuous datasets. This restricts its application to ordered hyperspectral images, a significant limitation in modern spectroscopic workflows where computational and memory constraints often make it infeasible to process large datasets in their entirety, forcing researchers to work with masked or extracted data subsets. Furthermore, common strategies to circumvent these limitations, such as patch-wise denoising, can introduce inconsistencies due to their reliance on localized noise models.

We have proposed a novel spatially invariant MNF (iMNF) method that overcomes this order-dependence by using a global, physically motivated noise model, calculated from the spectral “silent region”. We demonstrate that while standard MNF and iMNF perform comparably on spatially ordered data, only the iMNF method maintains its denoising capability when the data follows no order. This was visually confirmed by the preservation of key tissue morphology in the iMNF factors, which were completely lost in the standard MNF factors on shuffled data.

By proving its spatial invariance, our iMNF method expands the application of MNF denoising to a wider range of datasets beyond traditional images. This includes unstructured datasets, such as collections of single-point scans, where a PCA-like analysis is desired but can be significantly enhanced by the more sophisticated noise model that MNF provides. This could streamline research workflows by removing the need for time-consuming data reordering and enabling the analysis of previously incompatible datasets. Looking to the future, the strengths of both methods could be combined in a hybrid framework that dynamically assesses a dataset's structural order, allowing for an automated choice of best approach for data denoising. This ensures that the power of standard MNF is leveraged for well-ordered data, while the robustness of iMNF is applied where the assumption of local similarity does not hold. This could even take the form of a patch-wise iMNF implementation, which would combine a consistent, physically motivated noise model with a locally adaptive signal characterisation. Such an approach would not only offer a powerful tool for highly heterogeneous images but would also address the key issue of processing large hyperspectral datasets, which rapidly exceed the memory capacity of standard computing equipment. By removing the reliance on pixel adjacency, iMNF provides a solution to memory limitation problems that could face modern hyperspectral imaging. Large datasets, that otherwise may not be possible to load at once with standard computing equipment, can now be processed in independent patches without the risk of inconsistent noise models or edge artifacts.

To guarantee consistent denoising across these independent patches, a strict operational procedure must be followed. The global noise profile must be estimated only once. This can be derived from either a rapid calculation of the global mean, or from a representative spatial subset of data. This singular static noise covariance matrix is then applied unchanged to all subsequent patches. If estimated dynamically for each individual patch, the user risks reintroducing compositional dependent variations into the noise model.

Consequently, iMNF is universally applicable to both massive, memory-intensive images and unstructured, non-imaging datasets. Additionally, further memory efficiencies can also be gained by converting data from 64-bit to 32-bit floating-point precision, a strategy that halves the memory footprint with a typically negligible impact on spectral fidelity. Taken together, these advancements offer a robust framework for managing and denoising large-scale infrared data, removing a significant barrier to its widespread adoption in clinical research.

## Author contributions

Dougal Ferguson: conceptualization, methodology, data acquisition, formal analysis, investigation, visualization, writing – original draft, review & editing. Peter Gardner: conceptualization, writing – review & editing, supervision.

## Conflicts of interest

The authors declare that they have no known interests financial or personal that could have influenced the work reported in this paper.

## Ethics approval

The prostate cancer tissue microarray was accessed *via* the Manchester Cancer Research Centre Biobank (10_NOCL_02) under ethical approval granted by the South Manchester Research Ethics Committee (Ref: 22/NW/0237).

## Supplementary Material

AN-151-D6AN00152A-s001

## Data Availability

Data used in this study is freely available on Zenodo:^26,27^https://zenodo.org/records/18495062, and https://zenodo.org/records/18671069, with all code used in producing the results openly available on Github:^25^https://github.com/dougalferg/iMNF-denoising. QCL data used in the downstream ML assessment will be made available upon reasonable request to the authors. Supplementary information (SI) is available. See DOI: https://doi.org/10.1039/d6an00152a.
