# Supplementary material for: A spatially invariant noise model for minimum noise fraction (MNF) denoising of hyperspectral datasets: applications to large-scale infrared spectroscopic pathology
Source: Analyst. 2026 Mar 20;151(9):2624–37. doi: 10.1039/d6an00152a (PMC13015918; doi:10.1039/d6an00152a)
Supplement: AN-151-D6AN00152A-s001 [file AN-151-D6AN00152A-s001.pdf]

Supplementary Information (SI) for Analyst.
This journal is © The Royal Society of Chemistry 2026

Supplementary information to A Spatially Invariant Noise Model for Minimum Noise Fraction (MNF) Denoising of Hyperspectral Datasets: Applications to Large-Scale Infrared Spectroscopic Pathology.

Dougal Ferguson[ab], and Peter Gardner[ab*]

a. Department of Chemical Engineering, School of Engineering, University of Manchester, Oxford Road, Manchester, M13 9PL.

b. Photon Science Institute, University of Manchester, Oxford Road, Manchester, M13 9PL, United Kingdom.

* Corresponding author: peter.gardner@manchester.ac.uk

Savitzky-Golay parameter sweep

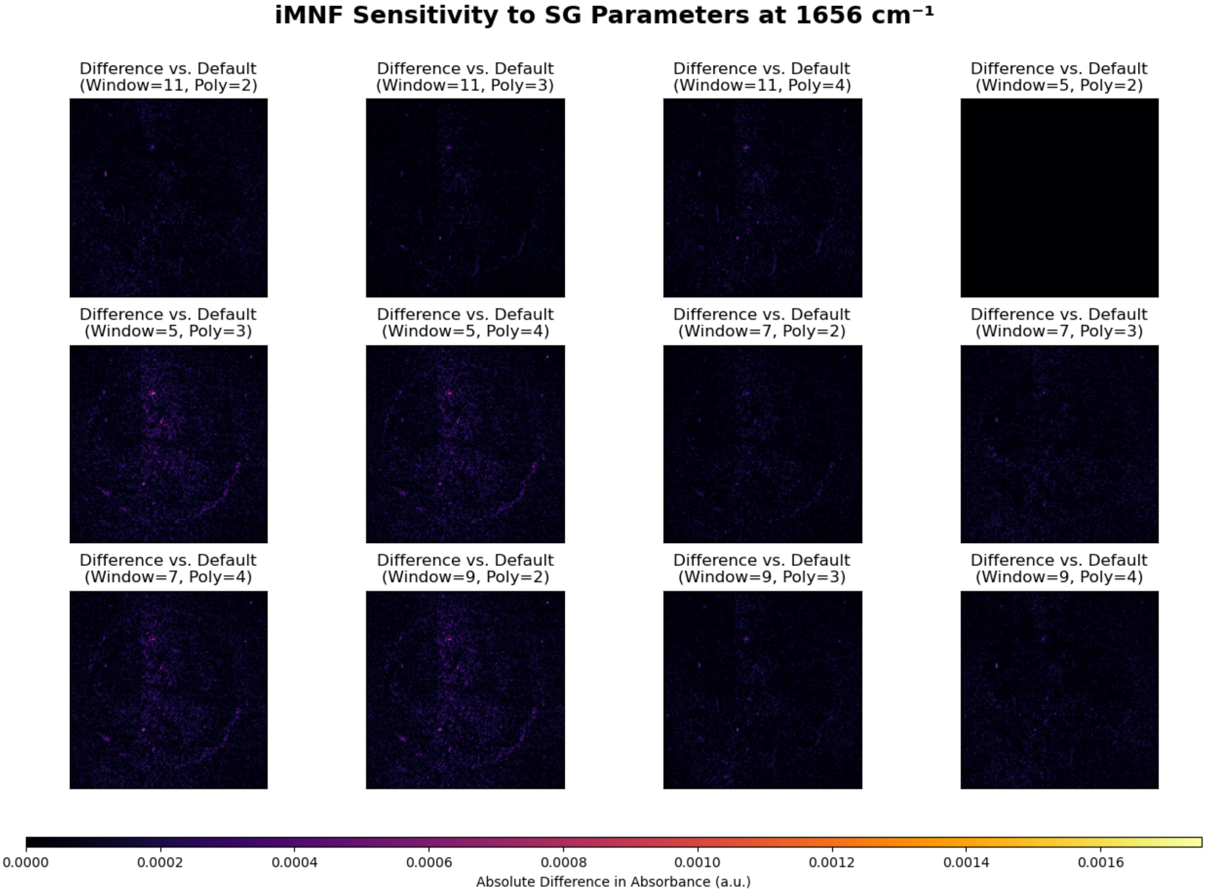

Figure 1 – Pixel-wise absolute difference at the Amide I band (1656 cm^{-1}) for differing Savitzky-Golay (SG) filter parameters for application of the iMNF denoising process. Robustness of the SG parameters is confirmed by the extremely small scale in absolute differences (2x10^{-3}).

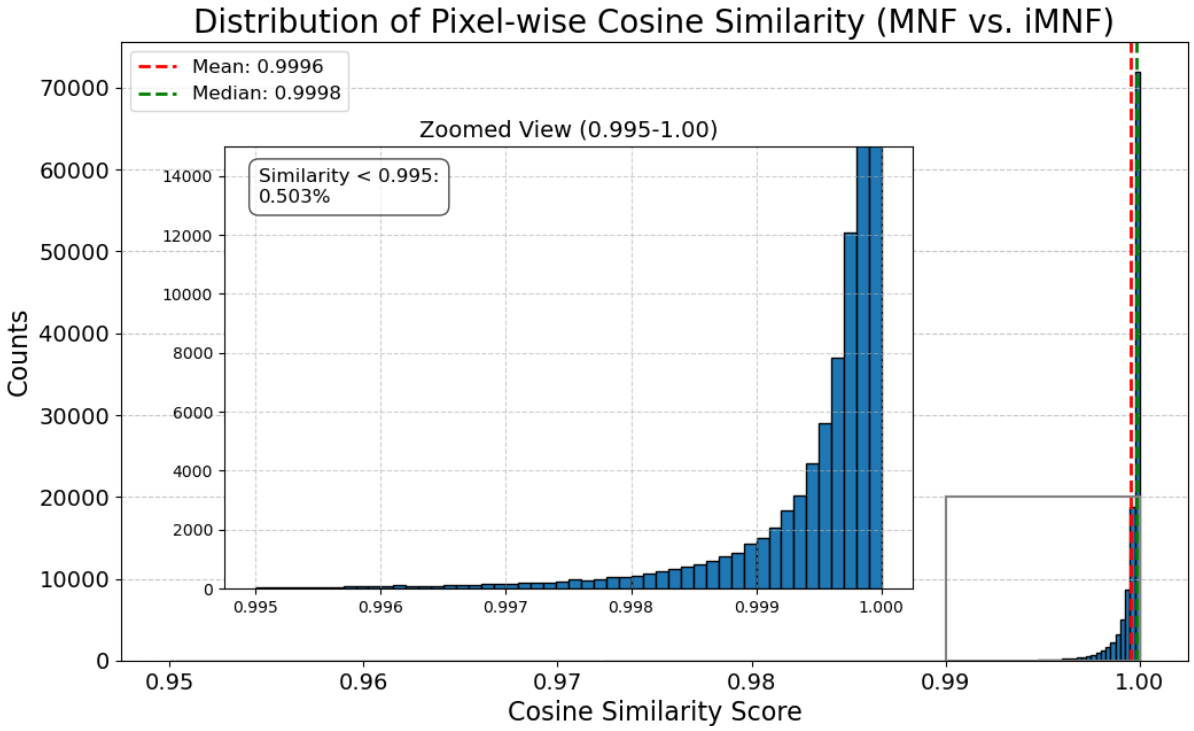

Figure 2 – Distribution of calculate cosine similarities between the denoised datasets from the MNF and iMNF algorithms. The high level of spectral similarity is shown by quantifying the percentage of cosine similarities that fall below a 0.995 value threshold.
